# Long-term follow-up after vertebroplasty – A mean 10-years follow-up control study

**DOI:** 10.1016/j.bas.2024.102783

**Published:** 2024-04-03

**Authors:** Fabian Cedric Aregger, Felix Gerber, Christoph Albers, Katharina Oswald, Christian Knoll, Lorin Benneker, Paul Heini, Ulrich Berlemann, Sven Hoppe

**Affiliations:** a Inselspital Bern, Berne University Hospital, Berne, Switzerland; b Wirbelsäulenmedizin Bern, Hirslanden Salem-Spital, Berne, Switzerland; c AO Foundation/ AO Innovation Translation Center, Dübendorf, Switzerland; d Orthopädie Sonnenhof, Berne, Switzerland

**Keywords:** Osteoporosis, Vertebroplasty, Trauma, Thoracolumbar

## Abstract

**Objectives:**

To evaluate the clinical 10 year outcome of patients treated with percutaneous vertebroplasty for vertebral compression fractures and to determine the incidence of new fractures in this time interval, as well as the mortality of the patients who underwent this procedure.

**Methods:**

All patients undergoing vertebroplasty for vertebral compression fractures between May 2007 until July 2008 were prospectively followed up at 10 years postoperatively. Patients were assessed for radiologic outcome and self-reported outcome parameters (PROs). Gathered parameters remained unmodified to the initial ones analyzing QoL improvement (EQ5D 3L and NASS score) and pain alleviation (VAS, NRS). Mortality was defined as an additional endpoint. Exclusion criteria include additional instrumentation, use of additional devices such as kyphoplasty balloons/stentoplasty, cognitive impairment, insufficient radiological documentation or absent re-consent.

**Results:**

Of 280 patients who underwent vertebroplasty, 49 (17.5%) were available for re-assessment with a mean follow-up of 10.5 years (9.9–11.1). Thirty patients (10.7%) were assessed clinically and radiologically, 16 (5.7%) in written form and three (1.1%) by phone only. A total of 186 (66.4%) died during the follow up period. Out of the remaining 45 patients, 27 patients declined participation, eight couldn't participate due to cognitive impairment, four had insufficient radiologic documentation. Six patients were lost to follow-up. At 10 years, patients reported a consistently improved quality of life (EQ-5D; p < 0.01) and global satisfaction. Vertebroplasty demonstrated a substantial and enduring effect on alleviating back pain over 10 years (p < 0.001). 26 (53%) patients experienced a new fracture since the initial procedure.

**Conclusion:**

A decade following vertebroplasty, patients continue to demonstrate a quality of life and pain level comparable to short and medium-term assessments, with a significant difference from baseline measurements. More than half (53%) of the patients participating at last follow-up experienced new fractures during this interim period. The cohort as a whole has been impacted by an elevated mortality rate over the time period.

## Highlights

• First study to publish mean 10-year results after vertebroplasty.
• Assessment of clinical outcomes and mortality in long-term follow-up.
• Recording of adjacent segment fractures over a long-term interval.

## 1 Introduction

Vertebral fractures constitute to the largest proportion among all osteoporotic fractures with an occurrence every 22 s in people over 50 years of age worldwide (Johnell and Kanis, 2006). Osteoporotic fractures are of socioeconomic relevance since >30% of women and 20% of men experience osteoporotic fracture in Western countries (Ballane et al., 2017).

Patients often suffer from local or radiating pain, biomechanical implications by post-traumatic deformity and secondary consequences such as cosmetic impairment with influence of self-perception potentially resulting in depression (Silverman, 1992). Since its introduction in the year 1984 by the pioneers Galibert and Deramond vertebroplasty has experienced a fluctuating history in terms of perception and acceptance in the medical community. Forty years after its first description, value of vertebroplasty for the treatment of pain and prophylaxis of progressive deformity is still controversial.

Despite being performed for almost four decades, there is a lack of long-term data available in the scientific literature on vertebroplasty. To our knowledge the longest follow-up study, including 100 vertebro-and kyphyoplasty patients, is five years (Liu et al., 2015). One case series of 11 patients reports on the outcome of patients treated with vertebroplasty for symptomatic Schmorl's nodes for an average of 58 (24–98) months (He et al.). The latest Cochrane review on vertebroplasty (Buchbinder et al., 2018) includes studies with a follow-up of up to 24 months.

In 2008, our group published a study on safety, efficacy and predictors for early reoperation in vertebroplasty. This prospective monocentric case series concluded that, “if routinely used, VP is a safe and efficacious treatment option for osteoporotic vertebral fractures with regard to pain relief and improvement of the QoL”. In 2018 this study cohort had its 10 year “anniversary”. Due to the lack of long-term data, we therefore examined the previously enrolled patients ten years after index surgery and defined the following objectives:

• to evaluate clinical outcome for patients treated with percutaneous vertebroplasty after 10 years assessed by patient related outcome measures (PROM)
• Determine the incidence of new fractures in this time interval
• Record mortality

## 2 Methods

We included every patient above 18 years treated with vertebroplasty at our institution between May 2007 and July 2008. In the context of the index study, the indication for vertebroplasty was established for patients who, despite receiving adequate analgesia, required hospitalization due to remaining non-mobilized for over one week. Patients with additional instrumentation, balloon kyphoplasty/stentoplasty, cognitive impairment and insufficient (initial) radiology were excluded. Patients were contacted at 10 years to assess radiologic and self-reported outcome parameters (PROs). Patients unable to attend personally were either interviewed by phone or contacted in written form. Death was determined as an additional endpoint considering the long follow-up period and advanced age of patients. Timing and cause of death were recorded.

Outcome parameters were collected on standardized scannable case report forms in the framework of the research program for the treatment of osteoporotic fractures of the Association for the Study of Internal Fixation (AO/AO-ASIF). Data was entered in the MEMdoc online database (http://www.memdoc.org) of the institute for Evaluative Research in Medicine (IEFM). The following documentation forms and outcome instruments were used: Euroqol-5D and NASS (North American Spine Society outcome assessment). Informed consent about participation had to be given by the patient as well as a completed Euroqol-5D, NASS and follow-up examination.

The patient cohort was split in patients participating at follow-up and non-participating to verify potential differences in baseline characteristics, preoperative comorbidities, initial surgery details, and preoperative patient reported outcomes (PRO). Patients with a 10 years follow-up were analyzed in descriptive manner regarding re-operation and vertebral fractures after initial surgery.

## 3 Statistical analysis

All continuous variables were summarized using the following descriptive statistics: n (number of valid observations), mean, standard deviation, median, maximum, and minimum. The frequency and percentages of observed levels were reported for all categorical measures. Comparison between groups for categorical data were performed using Chi-Square test or Fisher's exact test in case of spare data. Continuous data comparisons were performed using Wilcoxon rank sum test for not normal distributed data. EQ-5D index was calculated using the German TTO value set. Data cross tabulations were performed to compare individual preoperative comorbidities with those after 10 years follow-up. For repeated measurement analysis, we used an unadjusted mixed effect models with unstructured covariance to optimize the data which will be considered for the model. Furthermore, a descriptive sensitivity analysis was performed on patients who had preoperative and 10 years data available. Overall survival as well as fracture-free survival and reoperation-free survival were presented with Kaplan-Meier curves including 95% CI and patients at risk information. Patients who did not participate on the extension phase were censored at their last available study visit of the initial study.

Significance level was defined as p < 0.05. All statistical analyses were performed using SAS (version 9.4, SAS Institute Inc., Cary, NC, USA).

## 4 Results

**Fig. 1:**
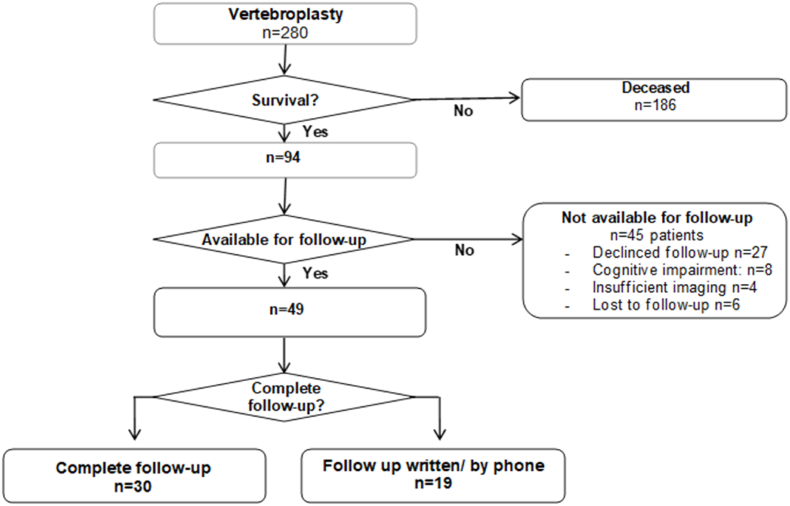
Flow-chart.

From May 2007 to July 2008, a total of 280 patients underwent vertebroplasty at our institution. Among this entire group, 186 (66.4%) patients died during the ten-year period of follow-up, leaving 94 (33.6%) patients. Out of the initial cohort of 94 patients, 45 individuals were excluded from the follow-up assessment for the following reasons: 27 patients (9.6%) declined to participate in the follow-up, eight patients (2.9%) were unable to undergo assessment due to cognitive impairment, and four patients (1.4%) had insufficient imaging data available. Additionally, six patients were deemed ‘lost to follow-up’ as they could not be contacted (as depicted in Fig. 1 Flow-Chart/Table 1).

**Table 1:** Overview about patient's status at 10 year visit (all patients).

Parameter	N = 280
Status at 10 years, n (%)	280
FU clinical	30 (10.7)
FU written	16 (5.7)
FU by phone	3 (1.1)
Passed away	186 (66.4)
Did not want to participate	27 (9.6)
Cognitive restriction	8 (2.9)
No x-rays available at all	4 (1.4)
Lost to FU	6 (2.1)

49 (17.5%) patients were available with a mean period of follow up of 10.5 (min. 9.9 – max. 11.1) years after index surgery. 30 (10.7%) patients were available for clinical and radiological assessment, 16 (5.7%) in written form and three (1.1%) by phone only.

Summarizing in groups of patients participating and non-participating results in 49 (17.5%) patients participating and 231 (82,5%) non-participating. Significant differences were observed between these two groups, with non-participating patients being notably older, with a median age of 79 years (p < 0.001) (Table 2). Patients participating at follow-up had a significant lower ASA status (p = 0.001). There was no statistically significant difference in the recorded number of fractures between the two groups, as both groups exhibited a mean of 2 fractures. Similarly, there was no significant disparity in the number of vertebroplasties performed, with four procedures in the participation group and five procedures in the non-participation group, respectively (Table 2).

**Table 2:** Demographics and disease details per 10 year status (all patients).

Parameter	Patient cohort at 10 years			*P* value
	Participation in extension study N = 49	No participation in extension study N = 231	Total N = 280	
Gender, n (%)	49	231	280	0.487^†^
Male	11 (22.4)	63 (27.3)	74 (26.4)	
Female	38 (77.6)	168 (72.7)	206 (73.6)	
Age (years) at surgery				<0.001^§^
n	49	231	280	
Mean (sd)	69.3 (7.8)	76.8 (9.2)	75.5 (9.4)	
Median (Q1; Q3)	69.4 (63.7; 75.1)	79.3 (71.7; 83.3)	77.2 (69.5; 82.4)	
Min; Max	54.1; 89.0	31.1; 94.0	31.1; 94.0	
Number of fractures (spine levels per patient)				0.631^§^
n	49	231	280	
Mean (sd)	2.0 (1.3)	1.9 (1.2)	1.9 (1.2)	
Median (Q1; Q3)	2.0 (1.0; 2.0)	2.0 (1.0; 2.0)	2.0 (1.0; 2.0)	
Min; Max	1.0; 6.0	1.0; 8.0	1.0; 8.0	
Number of vertebroplasty (spine levels per patient)				0.286^§^
n	49	231	280	
Mean (sd)	4.4 (1.9)	4.8 (1.6)	4.7 (1.7)	
Median (Q1; Q3)	4.0 (3.0; 6.0)	5.0 (3.0; 6.0)	5.0 (3.0; 6.0)	
Min; Max	1.0; 8.0	1.0; 11.0	1.0; 11.0	
ASA status, n (%)	45	209	254	0.001^‡^
I	3 (6.7)	4 (1.9)	7 (2.8)	
II	23 (51.1)	51 (24.4)	74 (29.1)	
III	19 (42.2)	148 (70.8)	167 (65.7)	
IV	0 (0.0)	6 (2.9)	6 (2.4)	
V	0 (0.0)	0 (0.0)	0 (0.0)	

Results
Patients reported outcome

### 4.1 Clinical outcome

The known significant improvement 8 weeks postoperatively remained significant after 10 years for EQ-5D (p < 0.01) (Fig. 4), NRS back pain (p < 0.01) (Fig. 5) and NASS lumbar pain (<0.001) (Fig. 6/Table 3/Table 4).

**Fig. 2:**
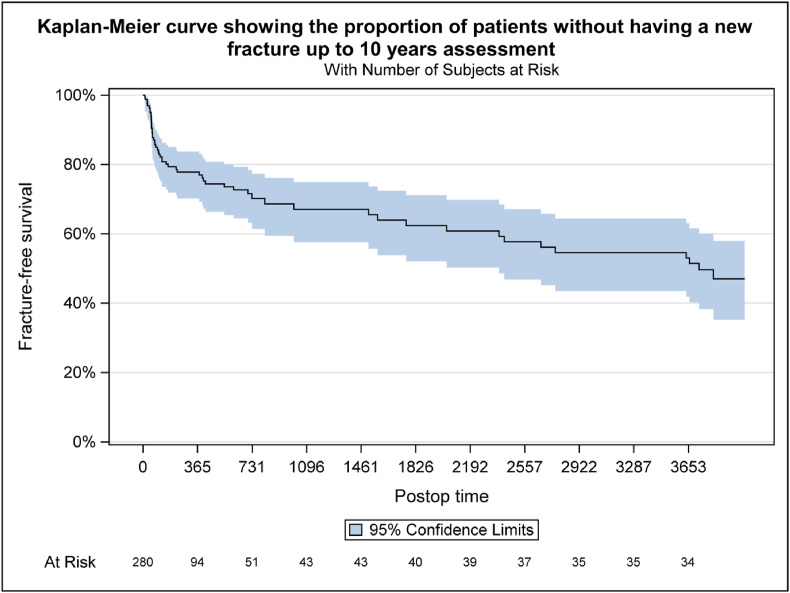
Note: Only fractures diagnosed/treated in Inselspital Bern were collected after 2 years assessment for patients who did not participate on 10 yrs extension. Note: For patients who occurred several fractures at different days during FU, only the first event was counted. Note: Patients who didn't have a new fracture confirmed are censored at the time of the last visit, which can be either 10 yrs visit or the last visit of the original study.

**Fig. 3:**
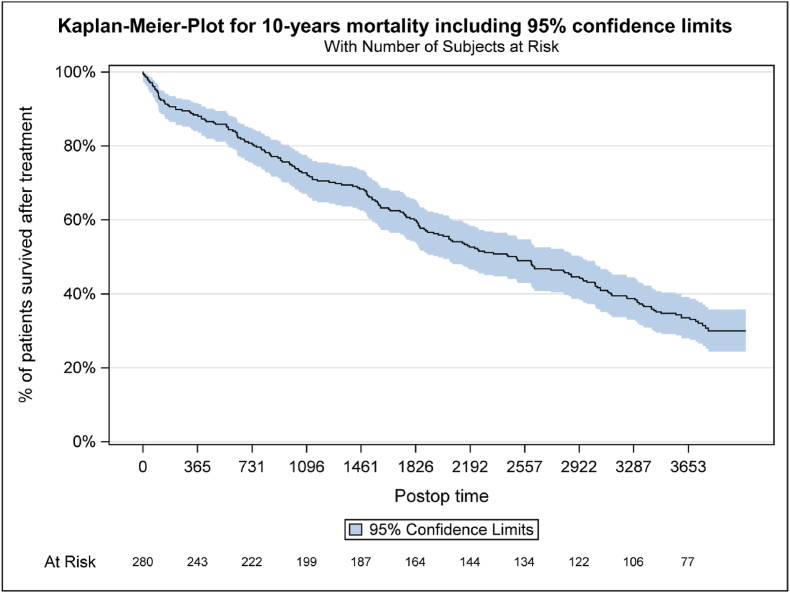
For patients with a known date of death, this date was used for the analysis. Patients who died between 2 and 10 years, for whom only the year of death was available, were imputed with the 30th June of the corresponding year. Patients who deliberately did not consent to the 10 year visit were marked as censored as at 31 December 2017. Patients lost to FU were censored at the time of their last study visit.

**Fig. 4:**
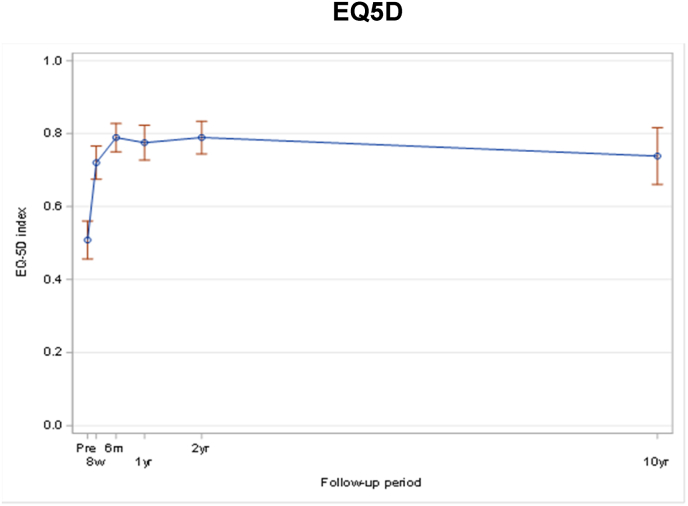
EQ5D.

**Fig. 5:**
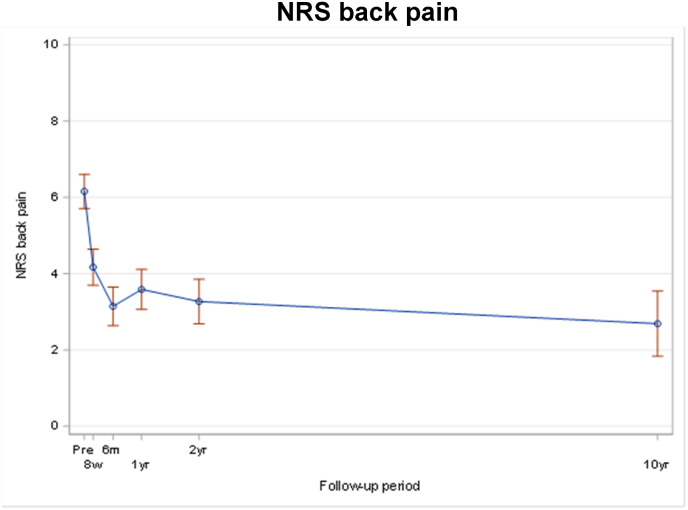
NRS back pain.

**Fig. 6:**
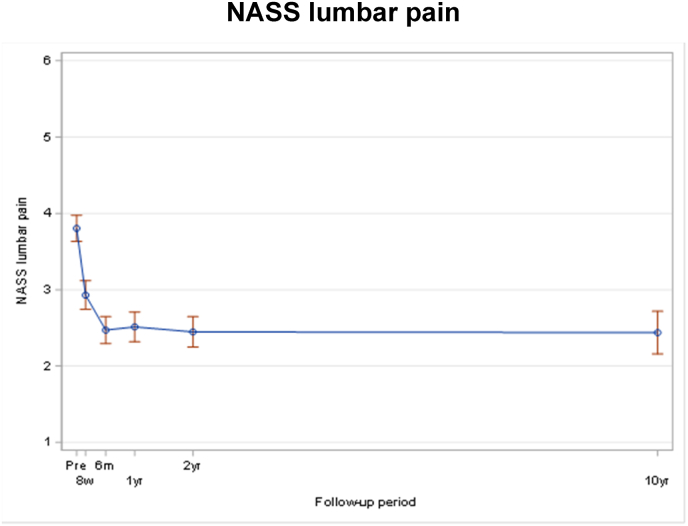
NASS lumbar pain.

**Table 3:** Pre-operative patient reported outcome per 10 year status (all patients).

Parameter	Patient cohort at 10 years			*P* value
	Participation in extension study N = 49	No participation in extension study N = 231	Total N = 280	
EQ-5D (3L) Index Score				0.368^§^
n	30	110	140	
Mean (sd)	0.56 (0.29)	0.50 (0.31)	0.51 (0.31)	
Median (Q1; Q3)	0.70 (0.20; 0.79)	0.70 (0.18; 0.70)	0.70 (0.18; 0.79)	
Min; Max	0.06; 0.90	−0.21; 1.00	−0.21; 1.00	
Back Pain NRS				0.934^§^
n	29	111	140	
Mean (sd)	6.3 (2.3)	6.1 (2.8)	6.2 (2.7)	
Median (Q1; Q3)	6.0 (5.0; 7.0)	6.0 (5.0; 8.0)	6.0 (5.0; 8.0)	
Min; Max	0.0; 10.0	0.0; 10.0	0.0; 10.0	
NASS: Lumbar Pain subscale				0.564^§^
n	29	111	140	
Mean (sd)	3.78 (0.89)	3.81 (1.06)	3.81 (1.03)	
Median (Q1; Q3)	3.82 (3.09; 4.36)	4.00 (3.27; 4.55)	4.00 (3.27; 4.45)	
Min; Max	1.91; 5.55	1.00; 5.64	1.00; 5.64

**Table 4:** Unadjusted mixed effect models derived estimates of the differences in mean scores by time measurements (all patients).

EQ-5D	n	Mean (95% CI)	Change (95% CI)	p-value^†^
Pre-operative	139	0.51 (0.46; 0.56)		
8 weeks	103	0.72 (0.68; 0.77)	0.21 (0.15; 0.28)	<0.001
6 months	102	0.79 (0.75; 0.83)	0.28 (0.22; 0.34)	<0.001
1 year	87	0.78 (0.73; 0.82)	0.27 (0.20; 0.33)	<0.001
2 years	78	0.79 (0.74; 0.83)	0.28 (0.22; 0.34)	<0.001
10 years - extension	49	0.74 (0.66; 0.82)	0.23 (0.14; 0.32)	<0.001
**Pain NRS back**	n	Mean (95% CI)	Change (95% CI)	p-value^†^
Pre-operative	139	6.2 (5.7; 6.6)		
8 weeks	109	4.2 (3.7; 4.6)	−2.0 (−2.6; −1.4)	<0.001
6 months	102	3.1 (2.6; 3.7)	−3.0 (−3.6; −2.4)	<0.001
1 year	88	3.6 (3.1; 4.1)	−2.6 (−3.2; −2.0)	<0.001
2 years	78	3.3 (2.7; 3.9)	−2.9 (−3.5; −2.2)	<0.001
10 years - extension	49	2.7 (1.8; 3.5)	−3.5 (−4.3; −2.6)	<0.001
**NASS lumbar pain**	n	Mean (95% CI)	Change (95% CI)	p-value^†^
Pre-operative	139	3.80 (3.63; 3.98)		
8 weeks	109	2.93 (2.74; 3.12)	−0.88 (−1.10; −0.65)	<0.001
6 months	102	2.47 (2.29; 2.65)	−1.33 (−1.55; −1.12)	<0.001
1 year	88	2.51 (2.32; 2.71)	−1.29 (−1.52; −1.06)	<0.001
2 years	78	2.45 (2.25; 2.65)	−1.36 (−1.59; −1.12)	<0.001
10 years - extension	49	2.44 (2.16; 2.72)	−1.37 (−1.67; −1.06)	<0.001

Follow-up intervals were allocated as following: ‘Pre-operative’ assessment before surgery, ‘8 weeks' assessment performed between 4 weeks and 10 weeks after surgery, ‘6 months' assessment performed between 4 months and 7 months, ‘1 year’ assessment performed between 10 months and 14 months, ‘2 years' assessment performed between 21.5 months and 26.5 months, and ‘10 years - extension’ between 9 years and 11.5 years after surgery. † P-value for comparison of change to pre-operative value.

Global satisfaction (NRS) remained at a high level until the ten years follow-up with a median of 7 at eight weeks, 8 at six months, 9 at one, two and ten years (Table 5).

**Table 5:** Patient satisfaction.

Parameter	Visit				
	8 weeks N = 98	6 months N = 102	1 year N = 86	2 years N = 77	10 years-extension N = 49
Global satisfaction NRS (patient)					
n	97	102	86	77	49
Mean (sd)	6.9 (2.3)	7.6 (2.3)	7.9 (2.3)	8.4 (2.1)	8.6 (1.8)
Median (Q1; Q3)	7.0 (5.0; 8.0)	8.0 (6.0; 9.0)	9.0 (7.0; 10.0)	9.0 (7.0; 10.0)	9.0 (8.0; 10.0)
Min; Max	0.0; 10.0	1.0; 10.0	0.0; 10.0	0.0; 10.0	2.0; 10.0
Comparison satisfaction, n (%)	98	102	86	77	49
Much better	39 (39.8)	52 (51.0)	47 (54.7)	45 (58.4)	27 (55.1)
Somewhat better	31 (31.6)	30 (29.4)	24 (27.9)	17 (22.1)	10 (20.4)
Same	8 (8.2)	12 (11.8)	8 (9.3)	12 (15.6)	10 (20.4)
Somewhat worse	1 (1.0)	5 (4.9)	1 (1.2)	3 (3.9)	0 (0.0)
Much worse	3 (3.1)	1 (1.0)	2 (2.3)	0 (0.0)	2 (4.1)
Too early to evaluate result	16 (16.3)	2 (2.0)	4 (4.7)	0 (0.0)	0 (0.0)

In the period from eight weeks to six months postoperatively, the percentage of patients reporting “much better” outcomes increased from 40% to 51%. Subsequently, this figure rose to 55% at one year, 58% at five years and 55% after ten years. The proportion of “somewhat better” outcomes was 32% at eight weeks and showed a gradual decline to 29% at six months, 28% at one year, 22% at two years, and 20% at ten years. The percentage of patients rating their condition as “same” was 8% at eight weeks, 12% at six months, 9% at one year and rose to 16% at two years and 20% at ten years. 1% reported a condition of “somewhat worse” at eight weeks, 5% at six months, 1% at 1 year, 4% at two years and 0% at ten years. Patients describing a clear deterioration as “much worse” constituted 3% at 8 weeks, 1% at six months, 2% at one year, and 4% at ten years (Table 5).

At the 10 years follow-up, 21 (70%) of the patients participating did not take any pain medication. Among the nine patients under medication, six (20%) took NSAID or paracetamol (WHO I), two (6.7%) strong opioids (WHO III); one (3.3%) took other pain medication (not recorded by the WHO analgesic scheme). Twenty-two (73.3%) were under osteoanabolic therapy.

### 4.2 New fractures

Within 10 years after index procedure 26 (53%) patients experienced new osteoporotic vertebral fractures (Fig. 2). 12 (24%) patients suffered from one, seven (14%) patients from multiple new fractures (maximum of four fractures). Ten (20%) patients required additional vertebroplasty, one (2%) with instrumentation. There was no significant difference in the incidence of adjacent and distal fractures.

### 4.3 Mortality

Mortality takes up by far the largest group of unreachable patients. In our collective, 186 (66.4%) deceased within 10 years after index procedure. The Kaplan-Meier-Plot (Fig. 3) showed a mortality rate of 30% at 4 years, 50% at 6,8 years and 66% at 10 years.

## 5 Discussion

Vertebroplasty is an established procedure with a turbulent history and of prevailing international acceptance regarding its safety (Cosar et al., 2009; Albers et al., 2019), effectivity in pain management and stability. Unsurprisingly there is heterogeneity regarding management of OVFs throughout recent publications. Missing long-term follow-up is one major point of criticisms in the literature (Halvachizadeh et al., 2021; Häckel et al., 2021).

This is, to our knowledge, the first monocentric case series of prospectively enrolled patients with a minimum follow up 10 years; a time-line of clinical relevance, since long-term results help us to justify indication for surgery and treatment costs.

Our data shows that all evaluated scores for quality of life and pain (EQ-5D, NRS back pain, NASS lumbar pain) as well as global satisfaction remain high until 10 years FU. Even though it is impossible to show a direct independent correlation between index procedure and reported outcome, it is evident that there is still a significant improvement in the parameters mentioned compared to baseline evaluation. While good short-term results were published already, the long-term effect of vertebroplasty on QoL and back pain remained unclear.

In our cohort, the proportion of patients rating their satisfaction as “much better” or “somewhat better” increased from 70% in the early postoperative period to 80% at one and two years, and remained at 75% after ten years. This also implies that 18% experienced no improvement after six months; 13% after one, 19% after two and almost 25% after ten years. A portion of this outcome can certainly be attributed to other factors causing back pain, yet the appropriateness of the indication for the procedure must also be critically evaluated. There is a definite risk for overtreatment in this context, which can also be demonstrated with regional variations (Scheuter et al., 2018). Nonetheless, the distribution of patient satisfaction in our cohort underscores that, despite ongoing controversies about and the potential placebo effect ascribed to it, vertebroplasty is effective (Clark and Diamond, 2023).

Apart from increased quality of life, global satisfaction and impact on back pain, long-term effects might also be reflected socioeconomically. Back pain is known to have a high socioeconomic burden at least since the 90s (Frymoyer and Cats-Baril, 1991). In this respect, the sustainable effect is also of economic relevance. Vertebroplasty and kyphoplasty are more expensive than conservative treatment short-term, but believed to be cost-efficient according to probabilistic analyses in mid and long-term outclinic patients, even if no mortality benefit is assumed (Hopkins et al., 2020).

There is no clearly proven, but repeatedly suspected connection between vertebroplasty and the occurrence of fractures of the adjacent vertebra. In our collective there was no difference between the occurrence of new fractures in adjacent or distant vertebrae. The fracture-free survival curve identified a higher risk within the first 6 months after surgery, whereas later the curve flattens. This might be explained by the delayed onset of new treatment and evolvement of osteoporosis therapy over the last years (Khosla and Hofbauer, 2017). There is evidence for the efficacy in fracture risk reduction in vertebral fractures for most approved treatments (Händel et al., 2023). In our collective at 10 years 47% experienced a fracture-free survival, 73% of which with osteoporosis treatment. The significance of osteoporosis therapy cannot be overstated, as represented by the increasing adoption of osteoanabolic treatments as a first-line option (Ferrari et al., 2020).

Latter is an important factor, since patients affected are known to have an increased mortality, which might be positively influenced by osteoporosis therapy (mumford 1993, 1993; Bolland et al., 2010). Our collective had a mortality rate of 66% in 10 years (30% at 4 years, 50% at 6,8 years and 66% at 10 years). Mortality rates up to 46% in 4 years (with a slight benefit over non-operative treatment (Edidin et al., 2015)) have been published. According to the federal bureau of statistics (Bundesamit für Statistik Lebenserwartung), the life expectancy at the age of 75 (median of our collective) in 2008 was 87.5 years with an expected mortality of 31% in 10 years. Even if the age distribution does not allow a direct comparison, it illustrates the significantly increased mortality in a collective that is often burdened with concomitant diseases, as reflected by the ASA score, which was significantly higher in the group non-participating. The ASA score has strong, independent associations with post-operative medical complications and mortality (Hackett et al., 2015). These fidings coincide with what is known from mortality associated with fragility fractures in other regions of the body and approaches the 10-year mortality from proximal femoral fractures (81.5%) (Galler et al., 2018). There is little evidence that vertebroplasty might reduce morbidity and mortality compared to non-surgical management (Cazzato et al., 2021). In our cohort the group affected by high mortality was significantly (exactly 10 years) older (mean 80 years) and more burdened in terms of health condition. Apart from age and ASA score both groups were comparable.

### 5.1 Our study has some limitations

110 patients (47%) of the patients non-participating in the 10 years follow-up did not fill out preoperative PROs.

it is quite possible that there is a certain blind spot of externally treated and in the meantime deceased patients. We tried to minimize that error by collecting as much information as possible by contacting the GP or family members.

Within the context of the index study, the duration between the onset of symptoms and the establishment of the indication was not recorded. Both the short-term and the long-term effects could be influenced by this.

Nevertheless, this study is the first of its kind to show the 10-year results after vertebroplasty. Even if the evaluation of the patients’ condition after the long-term course cannot be related independently to the intervention, it can be stated that no negative intervention associated consequences could be identified. After 10 years there was no relevant decline regarding back pain and quality of life compared to short-term results.

## 6 Conclusion

10 years after vertebroplasty, patients continue to demonstrate a quality of life and pain level similar to short- and medium-term outcomes, with a significant difference from baseline measures. More than half (53%) of patients participating at 10 years follow-up experienced a new fracture in the meantime. The collective is affected by an increased mortality rate over 10 years (66%).

## References

[bib1] Albers C.E., Schott P.M., Ahmad S.S., Benneker L.M., Nieuwkamp N., Hoppe S. (2019). Vertebral body lavage reduces hemodynamic response to vertebral body augmentation with PMMA. Global Spine J..

[bib2] Ballane G., Cauley J.A., Luckey M.M., El-Hajj Fuleihan G. (2017). Worldwide prevalence and incidence of osteoporotic vertebral fractures. Osteoporos. Int..

[bib3] Bolland M.J., Grey A.B., Gamble G.D., Reid I.R. (2010). Effect of osteoporosis treatment on mortality: a meta-analysis. J. Clin. Endocrinol. Metab..

[bib4] Buchbinder R., Johnston R.V., Rischin K.J. (2018). Percutaneous vertebroplasty for osteoporotic vertebral compression fracture. Cochrane Musculoskeletal Group. Cochrane Database Syst. Rev..

[bib5] Bundesamit für Statistik Lebenserwartung. https://www.bfs.admin.ch/bfs/de/home/statistiken/bevoelkerung/geburten-todesfaelle/lebenserwartung.html.

[bib6] Cazzato R.L., Bellone T., Scardapane M. (2021). Vertebral augmentation reduces the 12-month mortality and morbidity in patients with osteoporotic vertebral compression fractures. Eur. Radiol..

[bib7] Clark W., Diamond T. (2023). Early vertebroplasty for severely painful acute osteoporotic compression fractures: a critical review of the literature. Cardiovasc. Intervent. Radiol..

[bib8] Cosar M., Sasani M., Oktenoglu T. (2009). The major complications of transpedicular vertebroplasty: a review. J. Neurosurg. Spine.

[bib9] Edidin A.A., Ong K.L., Lau E., Kurtz S.M. (2015). Morbidity and mortality after vertebral fractures: comparison of vertebral augmentation and nonoperative management in the medicare population. Spine.

[bib10] Ferrari S., Lippuner K., Lamy O., Meier C. (2020). 2020 recommendations for osteoporosis treatment according to fracture risk from the Swiss Association against Osteoporosis (SVGO). Swiss Med. Wkly..

[bib11] Frymoyer J.W., Cats-Baril W.L. (1991). An overview of the incidences and costs of low back pain. Orthop. Clin. N. Am..

[bib12] Galler M., Zellner M., Roll C., Bäuml C., Füchtmeier B., Müller F. (2018). A prospective study with ten years follow-up of two-hundred patients with proximal femoral fracture. Injury.

[bib13] Häckel S., Renggli A.A., Albers C.E. (2021). How to measure the outcome in the surgical treatment of vertebral compression fractures? A systematic literature review of highly cited level-I studies. BMC Muscoskel. Disord..

[bib14] Händel M.N., Cardoso I., Von Bülow C. (2023). Fracture risk reduction and safety by osteoporosis treatment compared with placebo or active comparator in postmenopausal women: systematic review, network meta-analysis, and meta-regression analysis of randomised clinical trials. BMJ.

[bib15] Hackett N.J., De Oliveira G.S., Jain U.K., Kim J.Y.S. (2015). ASA class is a reliable independent predictor of medical complications and mortality following surgery. Int. J. Surg..

[bib16] Halvachizadeh S., Stalder A.L., Bellut D. (2021). Systematic review and meta-analysis of 3 treatment arms for vertebral compression fractures: a comparison of improvement in pain, adjacent-level fractures, and quality of life between vertebroplasty, kyphoplasty, and nonoperative management. JBJS Rev.

[bib17] He SC, Zhong BY, Zhu HD, et al. Percutaneous vertebroplasty for symptomatic Schmorl's nodes: 11 cases with long-term follow-up and a literature review. Pain Physician.:8. 28158154

[bib18] Hopkins T.J., Eggington S., Quinn M., Nichols-Ricker C.I. (2020). Cost-effectiveness of balloon kyphoplasty and vertebroplasty versus conservative medical management in the USA. Osteoporos. Int..

[bib19] Johnell O., Kanis J.A. (2006). An estimate of the worldwide prevalence and disability associated with osteoporotic fractures. Osteoporos. Int..

[bib20] Khosla S., Hofbauer L.C. (2017). Osteoporosis treatment: recent developments and ongoing challenges. Lancet Diabetes Endocrinol..

[bib21] Liu J.T., shun Li C., Chang C.S., Liao W.J. (2015). Long-term follow-up study of osteoporotic vertebral compression fracture treated using balloon kyphoplasty and vertebroplasty. J. Neurosurg. Spine.

[bib22] (1993). Mumford.

[bib23] Scheuter C., Wertli M.M., Haynes A.G. (2018). Unwarranted regional variation in vertebroplasty and kyphoplasty in Switzerland: a population-based small area variation analysis. PLoS One.

[bib24] Silverman S. (1992). The clinical consequences of vertebral compression fracture. Bone.

